# Dietary insulin index and load in relation to the risk of diminished ovarian reserve: a case–control study

**DOI:** 10.3389/fnut.2025.1559229

**Published:** 2025-05-22

**Authors:** Abed Ghavami, Sanaz Mehrabani, Mahdieh Khodarahmi, Amin Mokari-Yamchi, Mahdi Vajdi, Hatav Ghasemi-Tehrani, Gholamreza Askari

**Affiliations:** ^1^Department of Clinical Nutrition, School of Nutrition and Food Sciences, Isfahan University of Medical Sciences, Isfahan, Iran; ^2^Nutrition and Food Security Research Center, Isfahan University of Medical Sciences, Isfahan, Iran; ^3^Maternal and Childhood Obesity Research Center, Urmia University of Medical Sciences, Urmia, Iran; ^4^Department of Community Nutrition, School of Nutrition and Food Science, Nutrition and Food Security Research Center, Isfahan University of Medical Sciences, Isfahan, Iran; ^5^Department of Obstetrics and Gynecology, School of Medicine, Isfahan University of Medical Sciences, Isfahan, Iran

**Keywords:** dietary insulin index, dietary insulin load, diminished ovarian reserve, female infertility, reproductive health

## Abstract

**Background:**

Diminished ovarian reserve (DOR) occurs as a result of a decrease in the quantity and quality of oocytes, which can negatively affect fertility. Diet is one of the modifiable factors that plays an important role in preventing or exacerbating numerous diseases. As the effects of diet on the risk of DOR were not well-defined, this study was designed to investigate the association between DOR and dietary insulin index (DII) and dietary insulin load (DIL).

**Materials and methods:**

A total of 370 Iranian women participated in this case–control study: 120 individuals with DOR and 250 control subjects matched for age and body mass index. A validated semiquantitative 80-item food frequency questionnaire was used to assess the DII and DIL. Serum anti-Müllerian hormone levels were measured, the number of antral follicles was counted, and various anthropometric indices were evaluated. In addition, the relationship between the DII and DIL, and the risk of DOR was analyzed using multivariable logistic regression.

**Results:**

The unadjusted model of analysis found no significant relationship between the risk of developing DOR, and the DII and DIL. However, the findings showed that women who were in the highest quartile of the DII had a 1.29 times higher chance of having DOR (odds ratio: 1.29; 95% confidence interval: 1.07 to 3.93) when factors such as energy consumption and physical activity were considered. Furthermore, participants in the third and fourth quartiles of the DII and DIL had significantly higher odds of developing DOR when all possible confounders were taken into account.

**Conclusion:**

The risk of DOR increased with an increase in the DII and DIL. However, further clinical trials and prospective cohort studies are needed to support this finding.

## Introduction

Ovarian reserve refers to the total number of follicles at various stages of development within the female ovary, and their capacity to mature, develop, and be fertilized (1). The number of ovarian follicles in women decreases over time throughout their reproductive lifespan, eventually leading to suboptimal reproductive results (2). Diminished ovarian reserve (DOR) (3) is one of the leading factors contributing to infertility among women (4, 5). DOR is attributable to the decrease in the number of ovarian follicles and diminished oocyte quality, which can affect reproductive function and fertility in women (6). According to a study that pooled research data, the prevalence of infertility was 46.25% among women (7). DOR is often marked by lower levels of anti-Müllerian hormone (AMH), a reduction in the antral follicle count (AFC), and increased levels of follicle-stimulating hormone (8–10).

DOR can be attributable to various factors, including genetic predisposition (11), ovarian surgery (12), autoimmune diseases (13), and environmental influences (13). However, some DOR cases did not have a clear etiology and were classified as idiopathic (14). Since a significant number of women postpone their childbearing (15), understanding the influence of modifiable factors, such as dietary factors that affect ovarian reserve, is important (16). Dietary factors can influence the risk factors for DOR, including oxidative stress, inflammation, and insulin resistance (17–19). Increasing the insulin level leads to the senescence of granulosa cells of the ovaries by activating the NF-kB and ERK signaling pathways (18). Two dietary indices, dietary insulin load (DIL) and dietary insulin index (DII), were developed to assess the impact of insulin in foods and record the response to insulin in foods consumed, and these indices could be considered as risk determinants for developing insulin resistance (3, 20). The association of the DII and DIL with the odds of many health-related outcomes was assessed in previous studies (8, 21, 22). In addition, as reported in the literature, there exists a positive association between the DII and DIL, and the risk factors for DOR, including inflammation and obesity (8, 23). Studies have shown that obesity has negative effects on ovarian function by increasing insulin resistance and hyperinsulinemia accompanied by other mechanisms such as impairing gluconeogenesis (24, 25).

Only a limited number of studies have investigated the association between dietary indices and dietary patterns, and the risk of DOR (16, 26, 27). However, to the best of our knowledge, no study has evaluated the correlation between the DII and DIL, and the risk of DOR. A literature review has shown that the DII and DIL may influence ovarian reserve through several mechanisms, hence a case–control study among women who were referred to infertility clinics was conducted to evaluate this hypothesis.

## Method

### Participants

**Figure 1:**
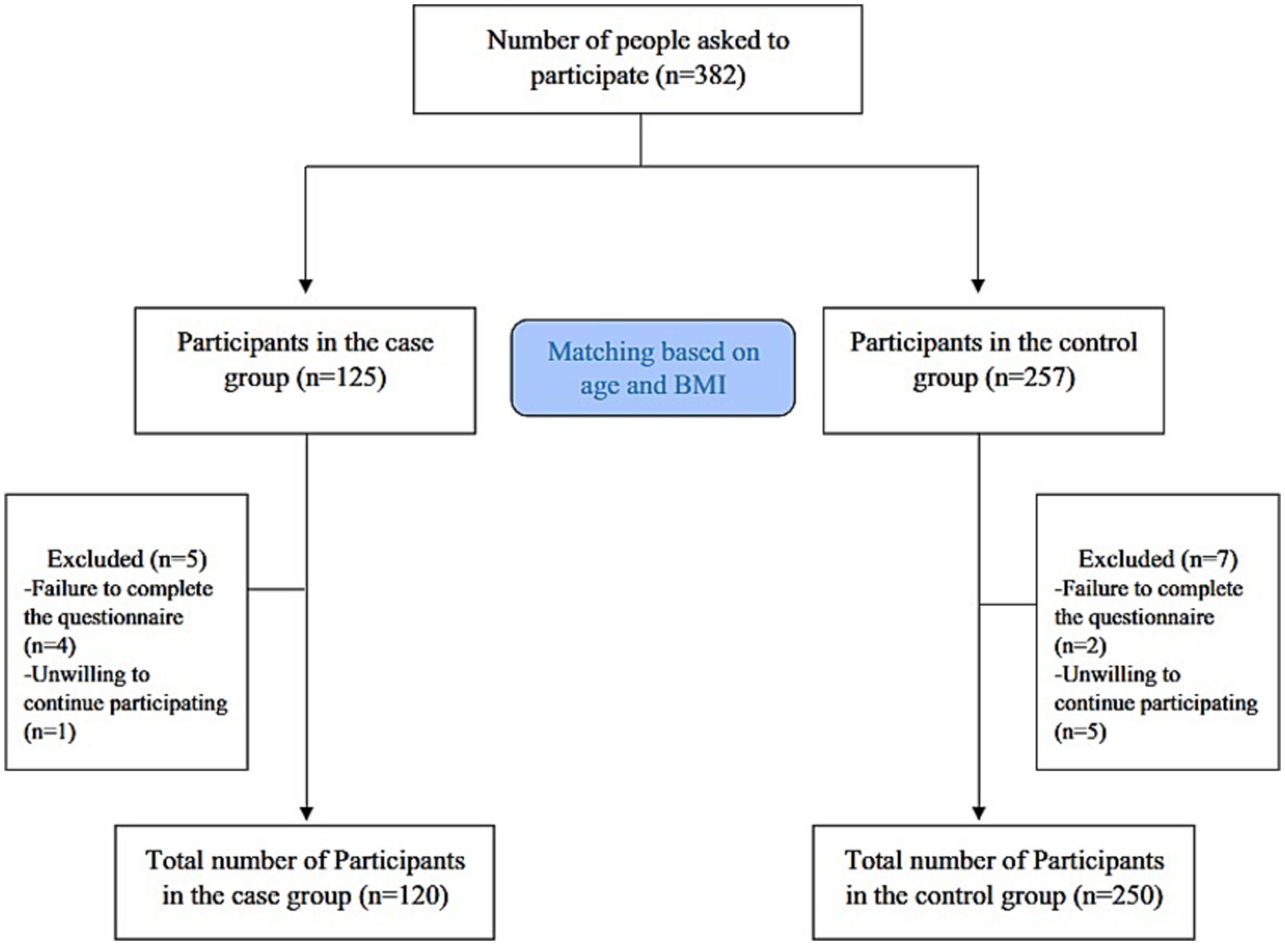
Flowchart of the study.

A total of 370 Iranian women were included in this case–control study, including 120 diagnosed with DOR (3) and 250 age- and body mass index (BMI)-matched controls. The participants were selected through purposive sampling from infertility centers affiliated with the Isfahan University of Medical Sciences, Isfahan, Iran. Briefly, women who met the following criteria were considered having been diagnosed with DOR and eligible cases. Transvaginal ultrasound and the diagnosis of DOR were made by a qualified gynecologist (H.GHT) based on either low AMH levels (≤ 0.7 ng/mL) or a low AFC (≤ 4 in both ovaries), or both (28). Women with a normal ovarian reserve during the same period from the same centers were randomly selected as controls. All participants were aged between 18 and 45 years, had a BMI ranging from 20 to 35 kg/m^2^, and were of Persian ethnicity. To ensure comparability, control subjects were matched with DOR cases based on age and BMI. A flowchart of the participant recruitment process is shown in Figure 1. The participants were categorized into age groups: under 25, 25–30, and over 30 years, and into BMI categories: under 24.9, 25–30, and over 30 kg/m^2^ (29). The sample size was calculated for this case–control study assuming two-tailed tests, 0.05 *α* level, 80% power, and a 2:1 control-to-case ratio. Based on the literature, approximately 30% of healthy women are exposed to a high DII/DIL (30, 31). An odds ratio (OR) of 2.0 was assumed for the association between high DII/DIL and DOR. Under these assumptions, the minimum required sample size was 111 cases and 222 controls (333 participants in total). Finally, by accounting for a 10% dropout, 370 participants were included in the present study.

Exclusion criteria included a history of ovarian surgery, chemotherapy, or radiotherapy, and conditions such as endometriosis, premature ovarian failure, and any endocrine and metabolic disorders. Participants on hormone therapy, special diets, or oral contraceptives in the 3 months prior to the study were also excluded. Written informed consent was obtained from all participants at the beginning of the study. This study was conducted based on the guidelines of the Declaration of Helsinki and was approved by the Ethics Committee of Isfahan University of Medical Sciences (IR.ARI.MUI.REC.1401.297).

### Dietary assessment

To evaluate the dietary intake of the participants, a validated semiquantitative food frequency questionnaire (FFQ) consisting of 80 food items was used (32). An expert dietitian conducted face-to-face interviews with all participants to complete the FFQ. Standard Iranian household measures were used to convert portion sizes into grams (33). In addition, the modified version of Nutritionist IV software for Iranian foods was used to estimate energy and nutrient intake (34).

### Assessment of DII and DIL

DII refers to the insulin response in the bloodstream after the consumption of each food item. It is calculated by dividing the area under the insulin response curve over 2 h after consuming a 1,000-kJ (239 kcal) portion of the test food by the area under the curve for a 1,000-kJ (239 kcal) portion of a reference food. The insulin index for the food items used in the FFQ was obtained from studies by Holt et al. (35), Bao et al. (36), and Bell et al. (37). For items whose insulin index was not available in the food list of the mentioned studies, the DII for similar food items was used. The following formula was used to calculate the insulin load of each food: insulin index of that food × energy content per 1 g of that food (kcal) × amount of that food consumed (g/d) (20). By summing the insulin load of each food, the DIL was obtained for each person. The DII was then calculated for each participant by dividing the DIL by the total energy intake.

### Anthropometric and laboratory assessments

A trained nutritionist measured all anthropometric values. The Seca scale was used to measure the weight and height of the participants while they were in a normal standing position, wearing light clothes with no shoes. BMI was calculated by dividing weight in kilograms by the square of height in meters. Waist circumference (WC) and hip circumference (HC) were measured to the nearest 0.1 cm using a tape. WC was measured between the lowest rib and the midpoint of the iliac crest, whereas HC was measured at the largest circumference around the buttocks. The waist-to-hip ratio (WHR) was calculated by dividing WC by HC. Body composition, including fat mass (FM) and fat-free mass (FFM), was evaluated via bioelectrical impedance analysis (BIA) using a body composition analyzer (Inbody 770, Inbody Co, Seoul, Korea). Blood pressure (systolic and diastolic blood pressure (SBP and DBP)) measurements were taken on the right arm while the participants were seated, using an automated digital sphygmomanometer (Microlife Blood Pressure Monitor A100-30, Berneck, Switzerland). The mean of these two readings was recorded as the participant’s blood pressure. In addition, physical activity levels were assessed using the validated Iranian version of the International Physical Activity Questionnaire (IPAQ) (38). Serum AMH levels were measured using the enzyme-linked immunosorbent assay (ELISA) method (Monobind, California, United States) following the manufacturer’s protocol. The minimum detectable concentration of AMH was 0.08 ng/mL.

Furthermore, a transvaginal ultrasound was performed to assess the AFC in both ovaries on the third day of an unstimulated menstrual cycle.

### Statistical analyses

Statistical analyses were conducted using SPSS (version 21.0, SPSS Inc., Chicago, Illinois, United States), with a two-tailed *p*-value of < 0.05, which was considered statistically significant. Participants were classified into quartiles based on DII and DIL scores. Chi-square test and one-way ANOVA were used to analyze categorical and continuous variables across these quartiles, respectively. Tukey’s honestly significant difference test was carried out after ANOVA performed multiple comparisons, to investigate the relationship between DII and DIL scores, and the risk of DOR. Multivariable logistic regression was carried out using two models with multiple covariates, such as FM, BMI, physical activity, weight, and total energy intake. Potential confounding variables were selected based on previous studies (16) and a directed acyclic graph (39).

## Results

This case–control study involved 120 women with DOR as the case group and 250 controls matched for age and BMI. Initially, 382 women were recruited. However, after the interviews, 12 women were excluded: 6 for not completing the questionnaire and 6 for unwillingness to continue participating in the study (Figure 1). DOR was identified through both AMH and AFC evaluations in 98 cases (81.66%), whereas it was determined solely using AFC measurements in 22 cases (18.3%).

The distribution of cases and controls based on selected socioeconomic and anthropometric variables is presented in Table 1. The mean BMI of cases and controls was 29.85 and 27.75 kg/m^2^, respectively. Compared with the control group, women in the case group had a higher mean level of FM (38.47 vs. 36.47, *p* = 0.02), WC (102.23 vs. 91.7, *p* = 0.002), and WHR (0.9 vs. 0.86, *p* = 0.003). In addition, serum AMH levels (0.56 vs. 4.11) and AFC (2.34 vs. 9.59) were significantly lower in the case group than in controls.

**Table 1:** Baseline characteristics of participants.

Variable		Case (*N* = 120)	Control (*N* = 250)	*p*-value*
Age (years)		33.37 ± 3.24	32.91 ± 3.15	0.196
BMI (kg/m^2^)		29.85 ± 2.49	27.75 ± 3.45	0.235
Weight (kg)		80.96 ± 4.78	79.26 ± 8.41	0.487
FM (kg)		38.47 ± 7.05	36.47 ± 8.91	0.020
FFM (kg)		57.99 ± 11.33	60.12 ± 11.97	0.098
WC (cm)		102.23 ± 35.95	91.70 ± 12.43	0.002
HC (cm)		109.10 ± 31.59	106.10 ± 11.57	0.316
WHR		0.90 ± 0.12	0.86 ± 0.08	0.003
SBP (mmHg)		122.18 ± 12.77	123.58 ± 14.03	0.341
DBP (mmHg)		79.41 ± 11.67	81.85 ± 10.48	0.056
Physical activity (MET/h/day)		19.05 ± 4.12	18.98 ± 4.51	0.896
SES (%)	Low	10 (8.3)	19 (7.6)	0.252
	Middle	50 (41.7)	127 (50.8)	
	High	60 (50)	104 (41.6)	
Education (%)	Illiterate	14 (11.7)	34 (13.6)	< 0.001
	≤ High school/diploma	31(25.8)	121 (48.4)	
	≥ College degree	75 (62.5)	95 (38)	
Occupation (%)	Housewife	82 (68.3)	184 (73.6)	< 0.001
	Employed	26 (21.7)	10 (4)	
	Student	12 (10)	56 (22.4)	
Previous pregnancy	Yes	99 (82.5)	203 (81.2)	0.441
	No	21 (17.5)	47 (18.8)	
AFC		2.34 ± 1.19	9.59 ± 2.24	< 0.001
AMH (ng/mL)		0.56 ± 0.71	4.11 ± 1.18	< 0.001

Quantitative variables are expressed as mean ± SD, and qualitative variables are expressed as *n* (%).

^*^p-values were from independent *t*-tests for quantitative variables and chi-square tests for qualitative variables between the two groups.

AFC, antral follicle count; AMH, anti-Müllerian hormone; MET, metabolic equivalent of task; BMI, body mass index; DBP, diastolic blood pressure; DOR, diminished or decreased ovarian reserve; FFM, fat-free mass; FM, fat mass; HC, hip circumference; SES, socioeconomic status; SBP, systolic blood pressure; WC, waist circumference; WHR, waist-to-hip ratio.

The general characteristics across the quartiles of the DII and DIL are presented in Tables 2, 3, respectively. As shown in Table 2, women with DOR showed significantly higher FM associated with increased DIL scores (*p* = 0.011). In addition, in the case group, a significant increase in BMI was observed across the quartiles of the DII score (*p* = 0.017). In the control group, BW also significantly increased across the quartiles of the DII score (*p* = 0.030). Furthermore, the percentage of previous pregnancies significantly declined in both groups, as shown in Table 3. Multivariable adjusted odds ratios (40) for DOR in the quartiles of the DII and DIL scores are provided in Table 4.

**Table 2:** Characteristics of participants according to the quartiles of dietary insulin load (DIL).

Variable		Case (120)					Control (250)				
		Q1	Q2	Q3	Q4	p*	Q1	Q2	Q3	Q4	p*
Age (years)		34.00 ± 3.45	33.06 ± 2.98	32.96 ± 3.32	33.59 ± 3.27	0.583	33.37 ± 2.95	33.33 ± 2.98	32.83 ± 3.51	32.17 ± 3.06	0.101
BMI (kg/m^2^)		29.39 ± 2.31	29.85 ± 2.26	30.41 ± 2.49	29.65 ± 2.93	0.439	27.12 ± 3.39	28.03 ± 3.46	27.78 ± 3.47	28.09 ± 3.47	0.357
Weight (kg)		81.57 ± 3.58	82.66 ± 4.12	83.00 ± 4.47	81.66 ± 4.14	0.545	78.35 ± 4.97	77.77 ± 5.31	78.37 ± 4.47	78.12 ± 4.95	0.902
FM (kg)		35.22 ± 4.33	39.97 ± 6.80	37.75 ± 5.54	40.87 ± 9.67	0.011	36.09 ± 8.5	37.75 ± 9.83	35.63 ± 8.66	36.53 ± 8.67	0.619
FFM (kg)		59.87 ± 11.84	56.75 ± 11.00	57.66 ± 11.76	57.95 ± 11.07	0.760	60.19 ± 12.80	60.07 ± 12.83	59.54 ± 10.71	60.60 ± 11.69	0.970
WC (cm)		98.03 ± 32.15	102.00 ± 36.50	98.71 ± 33.78	111.03 ± 41.55	0.516	92.7 ± 11.85	89.03 ± 14.10	92.15 ± 12.64	82.55 ± 11.27	0.338
HC (cm)		109.23 ± 31.91	115.03 ± 38.31	104.67 ± 26.33	106.96 ± 28.35	0.596	106.08 ± 10.55	103.92 ± 12.20	105.57 ± 12.87	108.38 ± 10.63	0.193
WHR		0.90 ± 0.0.098	0.91 ± 0.14	0.89 ± 0.13	0.90 ± 0.11	0.956	0.87 ± 0.08	0.85 ± 0.10	0.87 ± 0.06	0.85 ± 0.07	0.389
SBP (mmHg)		124.39 ± 11.73	120.39 ± 12.40	121.65 ± 14.23	122.70 ± 12.74	0.668	121.71 ± 13.94	124.55 ± 15.02	124.74 ± 14.39	123.60 ± 13.06	0.605
DBP (mmHg)		81.25 ± 11.91	77.90 ± 11.16	77.90 ± 9.46	81.14 ± 14.8	0.504	80.65 ± 11.23	82.50 ± 12.06	82.50 ± 12.06	81.91 ± 10.36	0.742
AFC		2.35 ± 1.25	2.42 ± 1.46	2.50 ± 1.31	2.03 ± 1.05	0.487	9.58 ± 2.27	9.55 ± 2.36	9.61 ± 2.08	9.61 ± 2.27	0.999
AMH (ng/mL)		0.73 ± 0.43	0.52 ± 0.22	0.49 ± 0.19	0.51 ± 0.21	0.551	4.09 ± 1.20	4.24 ± 1.18	3.91 ± 1.13	4.19 ± 1.22	0.458
Physical activity (MET/h/day)		19.53 ± 4.35	19.00 ± 3.97	19.75 ± 3.86	17.77 ± 4.23	0.276	19.23 ± 4.81	18.00 ± 4.09	19.86 ± 4.25	18.79 ± 4.67	0.155
SES (%)	Low	3 (10.7)	4 (12.1)	1 (3.1)	2 (7.4)	0.742	9 (13.4)	4 (7.1)	2 (3.4)	4 (5.9)	0.135
	Middle	11 (39.3)	14 (42.4)	16 (50)	9 (33.3)		36 (56.7)	30 (53.6)	29 (49.2)	30 (44.1)	
	High	14 (50)	15 (45.5)	15 (46.9)	16 (59.3)		20 (29.9)	22 (39.3)	28 (47.5)	34 (50)	
Education (%)	Illiterate	2 (7.1)	5 (15.2)	2 (6.3)	5 (18.5)	0.210	8 (11.9)	6 (10.7)	7 (11.9)	13 (19.1)	0.601
	≤ High school/diploma	9 (32.1)	9 (27.3)	11 (34.4)	2 (7.4)		37 (55.2)	28 (50)	29 (49.2)	27 (39.7)	
	≥ College degree	17 (60.7)	19 (57.6)	19 (59.4)	20 (74.1)		22 (32.8)	22 (39.3)	23 (39)	28 (41.2)	
Occupation	Housewife	19 (67.9)	24 (72.7)	19 (59.4)	20 (74.1)	0.596	52 (77.6)	40 (71.4)	40 (67.8)	52 (76.5)	0.750
	Employed	8 (28.6)	5 (15.2)	9 (28.1)	4 (14.8)		3 (4.5)	1 (1.8)	3 (5.1)	3 (4.4)	
	Student	1 (3.6)	4 (12.1)	4 (12.5)	3 (11.1)		12 (17.9)	15 (26.8)	16 (27.1)	13 (19.1)	
Previous Pregnancy	No	27 (96.4)	24 (72.7)	23 (71.9)	25 (92.6)	0.016	51 (76.1)	49 (87.5)	44 (74.6)	59 (86.8)	0.129
	Yes	1 (3.6)	9 (27.3)	9 (28.1)	2 (7.4)		16 (23.9)	7 (12.5)	15 (25.4)	9 (13.2)	

Quantitative variables are expressed as mean ± SD, and qualitative variables are expressed as *n* (%).

The SES score was evaluated based on the education level of both subjects and the family head, job of both subjects and the family head, family size, home status, and home type using a self-reported questionnaire.

AFC, antral follicle count; AMH, anti-Müllerian hormone; MET, metabolic equivalent of task; BMI, body mass index; DBP, diastolic blood pressure; DOR, diminished or decreased ovarian reserve; FFM, fat-free mass, FM, fat mass; HC, hip circumference; SES, socioeconomic status; SBP, systolic blood pressure; WC, waist circumference; WHR, waist-to-hip ratio.

^*^p-values were from independent *t*-tests for quantitative variables and chi-square tests for qualitative variables between the two groups.

**Table 3:** Characteristics of participants according to the quartiles of dietary insulin index (DII).

Variable		Case (120)					Control (250)				
		Q1	Q2	Q3	Q4	p*	Q1	Q2	Q3	Q4	p*
Age (years)		33.46 ± 3.35	35.26 ± 3.93	32.71 ± 2.92	33.29 ± 3.09	0.078	32.94 ± 2.88	32.97 ± 2.80	33.04 ± 3.78	32.57 ± 3.55	0.903
BMI (kg/m^2^)		28.03 ± 2.28	29.87 ± 2.25	30.24 ± 2.64	30.53 ± 2.48	0.017	27.40 ± 3.46	27.50 ± 3.46	27.88 ± 3.12	28.77 ± 3.05	0.184
Weight (kg)		81.93 ± 4.11	80.60 ± 2.87	82.71 ± 3.93	82.68 ± 4.46	0.317	76.94 ± 5.03	77.48 ± 5.97	78.80 ± 4.53	79.50 ± 2.29	0.030
FM (kg)		36.93 ± 4.99	37.29 ± 5.36	37.50 ± 6.21	40.01 ± 8.34	0.231	36.13 ± 9.82	36.58 ± 8.19	37.15 ± 8.99	36.21 ± 8.06	0.931
FFM (kg)		58.40 ± 10.14	57.78 ± 11.28	58.08 ± 11.62	57.86 ± 11.76	0.998	61.46 ± 12.76	58.78 ± 11.98	59.26 ± 10.91	60.69 ± 11.46	0.501
WC (cm)		97.33 ± 28.30	94.00 ± 28.46	108.84 ± 40.77	101.03 ± 36.03	0.491	91.43 ± 12.87	93.10 ± 12.91	90.75 ± 11.14	90.85 ± 12.25	0.695
HC (cm)		103.50 ± 25.89	111.46 ± 34.52	115.96 ± 38.72	104.80 ± 25.46	0.346	104.46 ± 11.12	108.25 ± 12.47	105.62 ± 10.73	106.32 ± 11.59	0.219
WHR		0.93 ± 0.08	0.87 ± 0.10	0.93 ± 0.14	0.87 ± 0.12	0.091	0.87 ± 0.08	0.86 ± 0.07	0.85 ± 0.07	0.85 ± 0.09	0.498
SBP (mmHg)		116.20 ± 12.65	125.86 ± 11.91	122.38 ± 10.35	122.70 ± 14.38	0.201	122.38 ± 14.20	123.44 ± 14.16	125.93 ± 15.21	123.62 ± 11.98	0.576
DBP (mmHg)		75.46 ± 12.97	84.0 ± 7.12	79.97 ± 10.39	78.80 ± 13.02	0.236	81.97 ± 11.25	80.87 ± 11.29	81.66 ± 10.83	83.62 ± 9.12	0.642
AFC		2.26 ± 1.03	2.73 ± 1.27	2.23 ± 1.26	2.33 ± 1.17	0.578	9.71 ± 2.21	9.64 ± 2.31	9.06 ± 2.05	9.85 ± 2.33	0.319
AMH (ng/mL)		0.93 ± 0.13	0.37 ± 0.14	0.54 ± 0.024	0.52 ± 0.19	0.161	3.90 ± 1.15	4.27 ± 1.16	4.20 ± 1.28	4.18 ± 1.13	0.210
Physical activity (MET/h/day)		20.20 ± 4.21	19.86 ± 3.96	18.64 ± 4.61	18.78 ± 3.75	0.508	19.40 ± 4.72	18.31 ± 4.30	19.20 ± 4.39	19.05 ± 4.58	0.468
SES (%)	Low	4 (26.7)	0	4 (10.3)	2 (3.9)	0.086	11(12.5)	4 (5.4)	2 (4.2)	2 (5)	0.333
	Middle	6 (40)	5 (33.3)	15 (38.5)	24 (47.1)		47(53.4)	38 (51.4)	24 (50)	18 (45)	
	High	5 (33.3)	10 (66.7)	20 (51.3)	25 (49)		30 (34.1)	32 (43.2)	22 (45.8)	20 (50)	
Education (%)	Illiterate	0	2 (13.3)	7 (17.9)	5 (9.8)	0.232	8 (9.1)	12 (16.2)	8 (16.7)	6 (15)	0.384
	≤ High school/diploma	5 (33.3)	6 (40)	11(28.2)	9 (17.6)		49 (55.7)	35 (47.3)	23 (47.9)	14 (35)	
	≥ College degree	10 (66.7)	7 (46.7)	21 (53.8)	37 (72.5)		31(35.2)	27 (36.5)	17(35.4)	20 (50)	
Occupation	Housewife	12 (80)	10 (66.7)	26 (66.7)	34 (66.7)	0.304	69 (78.4)	50(67.6)	33 (68.8)	32 (80)	0.441
	Employed	2 (13.3)	5 (33.3)	6 (15.4)	13 (25.5)		4 (4.5)	3 (4.1)	3 (6.3)	0	
	Student	1 (6.7)	0	7 (17.9)	4 (7.8)		15 (17)	21 (28.4)	12 (25)	8 (20)	
Previous pregnancy	No	14 (93.3)	13 (86.7)	32 (82.1)	40 (78.4)	0.575	63 (71.6)	63 (85.1)	41 (85.4)	36 (90)	0.034
	Yes	1 (6.7)	2 (13.3)	7 (17.9)	11 (21.6)		25 (28.4)	11 (14.9)	7 (14.6)	4 (10)	

Quantitative variables are expressed as mean ± SD, and qualitative variables are expressed as *n* (%).

The SES score was evaluated based on the education level of both subjects and the family head, job of both subjects and the family head, family size, home status, and home type using a self-reported questionnaire.

AFC, antral follicle count; AMH, anti-Müllerian hormone; MET, metabolic equivalent of task; BMI, body mass index; DBP, diastolic blood pressure; DOR, diminished or decreased ovarian reserve; FFM, fat-free mass, FM, fat mass; HC, hip circumference; SES, socioeconomic status; SBP, systolic blood pressure; WC, waist circumference; WHR, waist-to-hip ratio.

^*^p-values were from ANOVA for quantitative variables and chi-square test for qualitative variables across quartiles.

**Table 4:** Odds ratio (95% CI) of DOR according to the quartiles of dietary insulin index (DII) and dietary insulin load (DIL).

	DIL					DII				
	Q1	Q2	Q3	Q4	P-trend	Q1	Q2	Q3	Q4	P-trend
DOR/control	28/67	33/56	32/59	27/68		15/88	15/74	32/59	39/48	51/40
Crude	Ref (1.00)	0.95 (0.50–1.77)	1.02 (0.70–2.40)	1.17 (0.76–2.79)	0.819	Ref (1.00)	0.92 (0.64–3.39)	1.18 (0.87–3.51)	1.21 (0.95–3.58)	0.261
Model 1	Ref (1.00)	1.05 (0.62–2.43)	1.18 (0.81–2.61)	1.29 (0.90–3.49)	0.783	Ref (1.00)	1.07 (0.72–3.42)	1.21 (0.92–3.57)	1.29 (1.07–3.93)	0.015
Model 2	Ref (1.00)	1.18 (0.66–2.67)	1.42 (1.03–3.28)	1.63 (1.09–3.97)	0.031	Ref (1.00)	1.05 (0.68–3.43)	1.24 (1.05–3.59)	1.44 (1.09–3.95)	0.003

Multivariable logistic regression models were used with the adjustment for potential confounders. Model 1: physical activity + energy intake (kcal/day). Model 2: model 1 + fat mass (kg) and body mass index.

DOR, diminished or decreased ovarian reserve.

Post-hoc analysis showed that among all cases with DOR, FM (40.87 vs. 35.22) and FFM (59.87 vs. 57.95) were higher in the fourth quartile compared with the first quartile based on DIL scores. In addition, regarding DII scores, the analysis showed an increase in BMI (30.53 vs. 28.03), FM (40.01 vs. 36.93), and WC (97.33 vs. 101.03) in the highest quartile compared with the lowest quartile, whereas WHR decreased (0.87 vs. 0.93).

In the crude model, no significant relationship was observed between the risk of DOR and DII and DIL scores. After controlling for physical activity and energy intake in model I, women in the highest quartile of the DII score were 1.29 times more likely to have DOR (95% CI: 1.07–3.93). In model II, after controlling for physical activity, energy intake, FM, and BMI, the odds of DOR were significantly higher in the third and fourth quartiles of DII (OR: 1.24; 95% CI: 1.05–3.59 and OR: 1.44; 95% CI: 1.09–3.95, respectively) and DIL (OR: 1.42; 95% CI: 1.03–3.28 and OR: 1.63; 95% CI: 1.09–3.97, respectively).

## Discussion

To the best of our knowledge, the present investigation provided the first evidence on the association of the DII and DIL with the risk of DOR. Our results showed that a high-insulinemic diet was associated with a higher risk of DOR, with an increasing trend, especially in the maximally adjusted model. However, neither AMH levels nor AFC was associated with these dietary insulin indices.

It is evident that women with DOR have a high probability of being affected by reduced fecundability and poor reproductive outcomes, including premature menopause, an increased risk of miscarriage, and a failure to respond adequately to ovarian stimulation (41, 42). Although growing evidence has indicated that both oocyte quantity and quality may be affected by numerous factors such as age, exercise patterns, stress, smoking behavior, and genetic alterations (1, 43, 44), potential effects of diet on the indicators of ovarian reserve are scarce (16). Although there is no existing research investigating the association between dietary insulin indices and ovarian reserve to interpret our results, several studies have attempted to correlate nutritional components with fertility and evolution (45). For instance, a prospective analysis of the Nurses’ Health Study II (NHSII) yielded a direct association of total carbohydrate intake and dietary glycemic load with ovulatory infertility among healthy women with no history of infertility (46), whereas replacing dietary animal proteins with more plant-based proteins was strongly associated with a lower risk in this population (47). Similarly, a recent study by Eskew et al. has reported that higher compliance with a pro-fertility diet, which was characterized by high consumption of whole grains, seafood, soy foods, fruits, and vegetables with low pesticide residues and supplemental vitamin D, folic acid, and vitamin B12, was related to some improvements in the markers of ovarian reserve among overweight and obese women (16). Furthermore, a systematic review by Moslehi et al. has supported the view that serum 25-hydroxyvitamin D levels and the intake of soy or soy products can potentially influence ovarian reserve (45).

Although we could not find any significant associations between AMH levels and AFC—the most sensitive and specific markers of ovarian reserve quantification—and dietary insulin indices, there is convincing evidence suggesting that insulin resistance and hyperinsulinemia are crucial factors leading to endocrine dysfunctions and reproductive abnormalities such as ovulation disorders (48). Nonetheless, a recent prospective study reported inverse relationships between the dietary intake of carbohydrates, particularly dairy-based ones, fat, protein, and calcium from dairy sources, and the odds of a rapid reduction in AMH levels (49). Conversely, a systematic review of seven interventional studies revealed that low-carbohydrate diets are effective in resuming ovulation to boost pregnancy rates, particularly among overweight and obese women with polycystic ovary syndrome (50). Similarly, some studies have revealed the indirect favorable effects of low-glycemic-index carbohydrates on fertility and ovulation through hormonal regulation, which contributes to the normal ovulation process (51).

Despite the lack of significant associations between the DII/DIL and AMH, studies have demonstrated that ovarian aging and menopause, the final distinctive feature of DOR, are strongly related to the risk of cardiovascular diseases (CVDs) due to the diminished production of estrogen from the ovaries. Interestingly, it has been claimed that reduced AMH levels may contribute to cardiovascular decline independent of the role of estrogen. However, studies available in this regard are inconclusive, particularly those with an observational design (52). The data obtained from a cohort study of Iranian premenopausal women showed less favorable cholesterol and low-density lipoprotein profiles among those with a lower baseline age-specific AMH quartile over a follow-up time of 12 years (53). Another longitudinal study revealed an independent effect of the AMH level on the occurrence of coronary heart disease and CVDs (54). Nevertheless, such an association is still questionable regarding metabolic syndrome and some of its components (55). Hence, further research is required to clarify these probable associations before considering them in clinical practice.

Considering the absence of published literature evaluating the relationship between the DII/DIL and ovarian reserve, elucidation of our results is challenging. However, the metabolic relevance of dietary insulin indicators has been documented in numerous previous investigations (8, 23, 56, 57). In a previous study, a cross-sectional analysis of 8,691 adult participants showed that following a diet with a high DIL and DII enhances the risk of general obesity in women (8). A similar relationship was found in a cross-sectional analysis of data from the Shahedieh cohort study that proposed a robust association between the DII/DIL and metabolic syndrome risk in women (58). In contrast, there were several observational studies that did not observe unfavorable effects of higher scores of these indicators on various chronic conditions (59). These inconsistencies may be attributable to the use of various methods for evaluating insulinogenic effects of foods; diverse food processing and cooking techniques, which differ across various countries; and controlling for disparate confounding variables in analyses.

The potential underlying mechanisms in the association between ovulation disorders and the DII/DIL are not fully known. However, it has been hypothesized that the impact of insulinogenic foods, which directly increase insulin secretion, on tissue sensitivity to insulin may be responsible for this detrimental ovulation status (60). In other words, insulin, by stimulating the response of ovarian follicles to gonadotropin, contributes to developing anovulatory infertility (61). In addition, studies have reported a strong correlation between hyperinsulinemia and hyperandrogenism, which can eventually lead to ovulation disorders (62). Oxidative stress and low-grade inflammation by the production of reactive oxygen species are other mechanisms that are involved in the association between DOR and the diet’s insulinemic potential (63). In this regard, carbohydrate-rich diets, particularly those with a high glycemic index, a high fructose content, and a low fiber content, have been shown to promote a greater proinflammatory state (63). Intriguingly, recent experimental and clinical trial studies have confirmed the protective effects of antioxidant compounds against oxidative damage to ovarian reserve (64–66). Furthermore, higher insulin secretion in response to a diet with high insulinemic potential may eventually result in increased fat storage as insulin lowers fat oxidation (67). On the other hand, postprandial hyperglycemia induced by insulinemic foods, which have a high rate of digestion and absorption, can lead to a decline in glucose excursion and subsequently reduction in satiety, restoring hunger sensation, excessive food intake, and subsequent increment in adiposity (68). It should be noted that obesity may exacerbate the ovarian reserve status, as it has been reported that obese women have a lower AMH level than normal-weight women (69).

This study has several strengths that should be mentioned. It investigated the relationship between the DIL/DII and DOR for the first time in women. Other strengths include a large sample size, matching cases and controls based on age and BMI, using a reliable FFQ for dietary evaluation, and adjusting for a broad variety of potential confounders in our analysis. However, several limitations ought to be considered while interpreting our findings. First, the retrospective nature of this case–control study prevents us from inferring a causal association between the DII/DIL and DOR. Second, as some food items in the FFQ were not accessible in the database, the DII values of similar foods were used, which may introduce a bias in the calculation of the insulinemic potential of diets. Third, despite adjusting for several confounders, residual confounding effects from variables such as psychological and genetic factors and other unmeasured confounders may attenuate the estimated independent associations. Fourth, as this study was carried out among women attending infertility clinics, the findings may not be generalizable to women in the general population. Last, despite the usage of valid and reliable questionnaires, similar to all epidemiological studies, some degree of measurement error was inevitable.

## Conclusion

In conclusion, the results of the present study provide evidence suggesting that adherence to a diet with a high insulin index and load may be associated with an increased risk of DOR, whereas no significant association was revealed between the insulinemic potential of a diet and the markers of ovarian reserve (AMH levels and AFC). Our findings may be beneficial for developing new dietary recommendations for women with DOR, but they need to be confirmed in future research. Given the scarcity of data on this topic, further studies, especially prospective studies and randomized trials, are warranted to shed light on this subject.

## Data Availability

The datasets analyzed during the study are available from the corresponding author on reasonable request.
